# Collision sensitive niche profile of the worst affected bird-groups at wind turbine structures in the Federal State of Brandenburg, Germany

**DOI:** 10.1038/s41598-018-22178-z

**Published:** 2018-02-28

**Authors:** Anushika Bose, Tobias Dürr, Reinhard A. Klenke, Klaus Henle

**Affiliations:** 1 Department of Conservation Biology, UFZ – Helmholtz Centre for Environmental Research, Permoserstraße. 15, D-04318 Leipzig, Germany; 2 Brandenburg State Agency for Environment, Brandenburg State Bird Conservation Centre, Unit N3, Buckower Dorfstraße 34, 14715 Nennhausen/OT Buckow, Germany

## Abstract

Biodiversity-related impacts at wind energy facilities have increasingly become a cause of conservation concern, central issue being the collision of birds. Utilizing spatial information of their carcass detections at wind turbines (WTs), we quantified the detections in relation to the metric distances of the respective turbines to different land-use types. We used ecological niche factor analysis (ENFA) to identify combinations of land-use distances with respect to the spatial allocation of WTs that led to higher proportions of collisions among the worst affected bird-groups: Buntings, Crows, Larks, Pigeons and Raptors. We also assessed their respective similarities to the collision phenomenon by checking for overlaps amongst their distance combinations. Crows and Larks showed the narrowest “collision sensitive niche”; a part of ecological niche under higher risk of collisions with turbines, followed by that of Buntings and Pigeons. Raptors had the broadest niche showing significant overlaps with the collision sensitive niches of the other groups. This can probably be attributed to their larger home range combined with their hunting affinities to open landscapes. Identification of collision sensitive niches could be a powerful tool for landscape planning; helping avoid regions with higher risks of collisions for turbine allocations and thus protecting sensitive bird populations.

## Introduction

Global environmental change strongly impacts the structure of biological communities^1,2^ leading to accelerated biodiversity loss. There is an increasing concern about the negative effects of climate change on biodiversity, ecosystem services, and human society as a whole^3^. Concerns about the impacts of climate change on society have triggered shifts in energy systems of several countries, among them Germany, with a high investment in the renewable energy sector^3,4^. The expansion of renewable energy is a central element of the German Federal Government’s climate and energy policy. The target for 2020 is to produce 30% of the electricity from renewable energies^5^. Particularly wind energy is increasingly explored as an alternative energy source, leading to the widespread construction of wind farms.

On the other hand, this growing production of wind energy is accompanied by the emergence of new conservation issues; in particular, the collision of birds and bats through direct impacts with the turbine structures^6,7^. Additionally, the indirect effects of the loss of nesting and foraging habitats add on to the concerns mentioned above^8^. Therefore, environmentalists and managers have argued against the installation of wind farms in areas with high densities of birds^9^. They make the simplistic assumption that the higher the abundance of individuals of a given species at a particular site, the higher is their susceptibility to collisions with wind energy structures installed at that particular site^10^. This assumption has been readily challenged by many researchers, since their findings show that the pre-construction bird abundances and the observed numbers of carcasses as a measure of post-construction bird collisions through detections are not closely related^10,11^.

In order to resolve this contradiction and to correctly guide the installation of future wind farms several researchers have tried to assess the effects of wind farms on wildlife by monitoring collisions after the construction of wind turbines (WTs)^10–12^. These long-term detections are based on carcass search operations conducted around the turbines. They underestimate the actual number of individuals being killed to a different degree due to a) spatial incompleteness related to non-uniformity in the searches, b) temporal incompleteness related to duration and periodicity of intervals between the searches, c) incomplete detection related to variability in carcass persistence time of birds of different sizes, and d) variation in detection probabilities related to the types of vegetation cover, substratum and the species involved in the searches^13^. These shortcomings limit the ability to compare sites and to determine the cumulative impacts of turbines on species as well^14^. However, there are studies that have accounted for some of these short-comings by correcting for detection biases^7,15^, or by comparing searcher efficiencies and carcass persistence times by trials using surrogate carcasses^13^. Few other studies have also highlighted the effects of landscape on the detected bird collisions, particularly of features around the locations of the WTs^16^. Our study changes the perception of this view to their spatial aspects and tries to highlight the effect of distances of WTs to habitat elements of different categories in the surrounding landscape. Distance values and thresholds to edges of habitat elements, e.g. special objects like nesting trees, are often required when policymakers ask for information ensuring safe deployment of WTs. The increase and decrease of the collision risk at distances in the immediate vicinity or away from these specific features can thereby propose safer placements of WTs in the landscape and identify areas where the risks of bird collisions could be minimized^17^.

**Figure 1:**
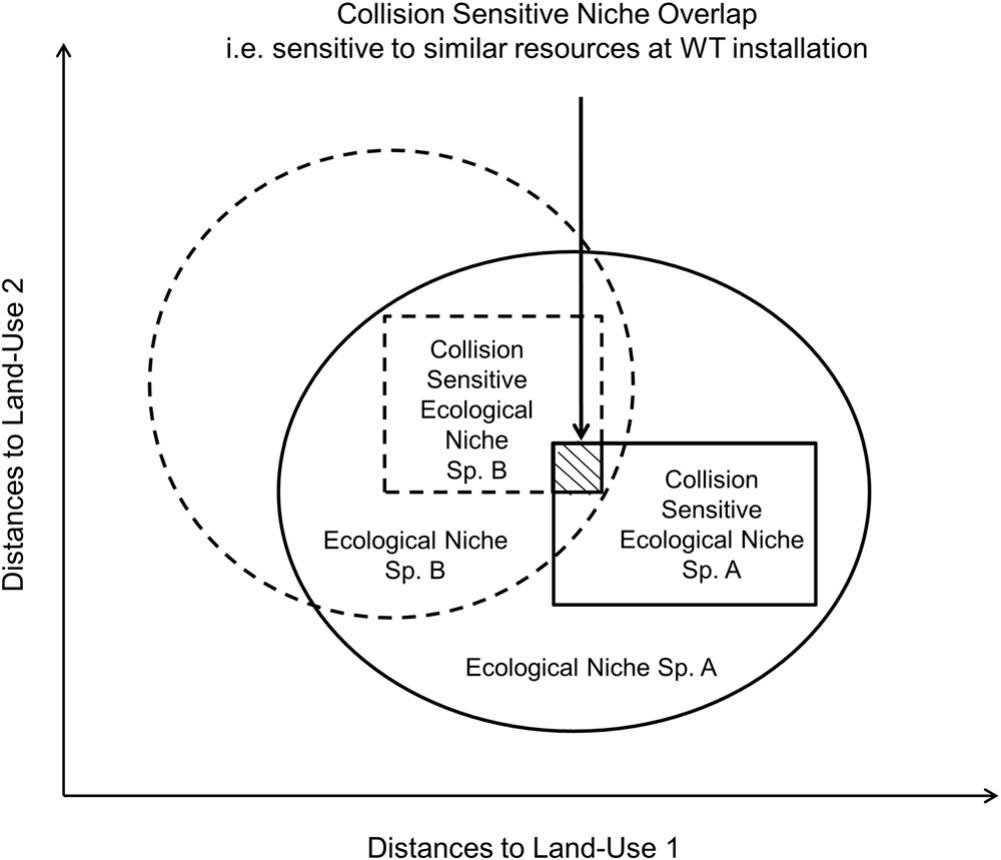
Thematic diagram explaining the collision sensitive ecological niche with respect to the ecological niche against distance to edge based land-use classes.

In response to similar concerns regarding the direct collision based impacts of wind farms on birds, we analyzed long-term carcass detections from monitoring operations in the state of Brandenburg, Germany, in relation to the local landscape. We evaluated the effects of distances between turbines and different land-use types on collision risks, specifically for the worst hit taxon related groups of birds in our sample, using the multivariate approach of Ecological Niche Factor Analysis (ENFA), which is based on Hutchinson’s *n*-dimensional hypervolume^18–20^. We ascertained their collision niche; a part of their fundamental ecological niche and obviously their realized ecological niche^21^, only representing a part inside their respective existent hyperspace that is influenced by the deployment of technical infrastructures causing potential collisions, thus referred to as the “collision sensitive ecological niche” (Fig. 1). We focused on assessing the similarities and dissimilarities between these collision sensitive niches of all the bird-groups under study, to enable the guidance of potential management interventions across multiple groups.

## Methods

### Study area

**Figure 2:**
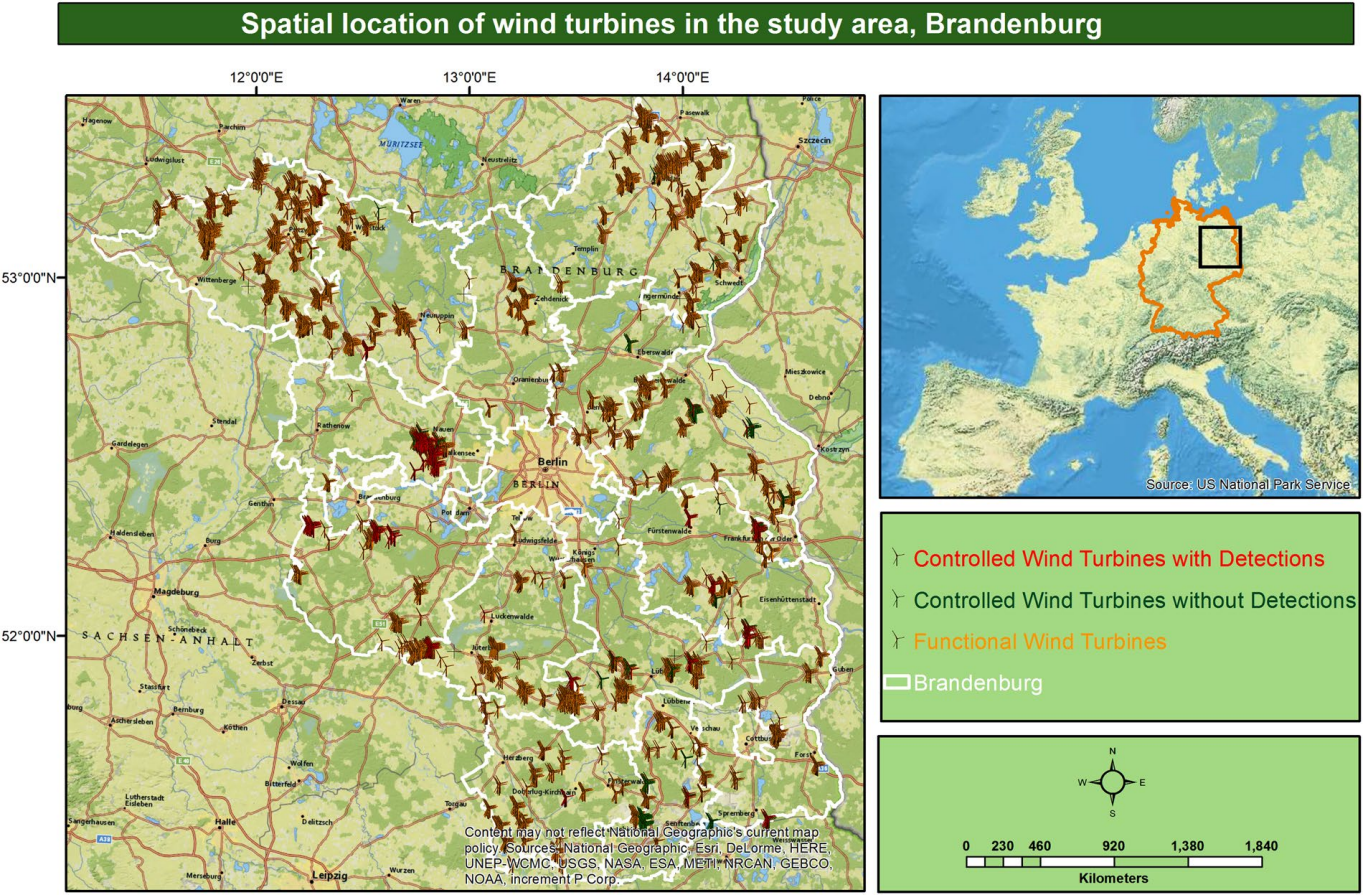
Study area showing the spatial locations of all the functional Wind Turbines surveyed (with and without carcass detections). Map source: *ESRI*. *ArcGIS Desktop: Release 10*.*1*. *Redlands*, *CA: Environmental Systems Research Institute*.

The study area, the Federal State of Brandenburg (Fig. 2), is located in the north-eastern part of Germany. It covers an area of approximately 29,500 km^2^, interspersed with around 27,000 km of rivers and around 3,000 lakes. Half of the state’s area is utilized by agriculture and for livestock raising and roughly another one-third of the region is covered by forests^22^. Over the past two decades, WT structures have contributed substantially to the landscape of Brandenburg, and have emerged as a new cause of bird loss^23^.

### Carcass search data

Carcass detections were spatially limited and available from 69 of the 3811 wind farms currently functional in the state, comprising of 617 turbines with rotor diameters varying from 40 m to100 m and nacelle heights varying from 41.5 m to 160 m. A total of 7428 search operations were made between 2000 and 2011 with around 1–31 (mean 8.1) turbines reportedly controlled per search, out of which only 450 searches detected bird carcasses. The time interval between these search operations (searching the same turbine) varied between 1 and 188 days with a median of 2 days (mean 5.3 days)^7^.

The data were collected either in special monitoring surveys following the construction of a wind park (carcass searching) as requested by the authority responsible for permission to construct wind parks according to the German nature conservation and planning law on federal and state levels. Further data provided to this database were based on single sampling actions e.g. either by the state agency mentioned above and only a few from collision victims accidentally found by private people during a walk or other leisure activities on their own property or on public land.

More information about the sampling can be found at: http://www.lugv.brandenburg.de/cms/detail.php/bb1.c.312579.de.

We know about the problems related to species-specific carcass persistence, searcher efficiency, and substratum or vegetation cover^13^. However, because it is not only difficult and partly also impossible to account for this and to standardize the data, we used a rather conservative approach neglecting the detailed but often very biased information. Instead, we solely utilized the respective spatial information of the detected carcasses to ascertain the combination of predictors influencing the collision phenomena at the spatial location of the particular turbines where the bird carcasses have been detected.

The general assumptions we follow in this paper are the following:

- the allocation of WTs in a certain distance to habitat elements (land-use factors) and the combination of factors may have an influence on the probability to collide,
- other factors, that are independent of the special allocation mentioned in (a) like, e.g. the influence of the season, are ignored. We don’t make any stratification regarding this,
- turbines, that have been controlled during the study, but had never shown any collisions, are used as a controls,
- the whole analyses are based on the binary information of turbine sites without any fatalities and those where one or more fatalities belonging to one or more bird-groups considered in this study were found.

**Figure 3:**
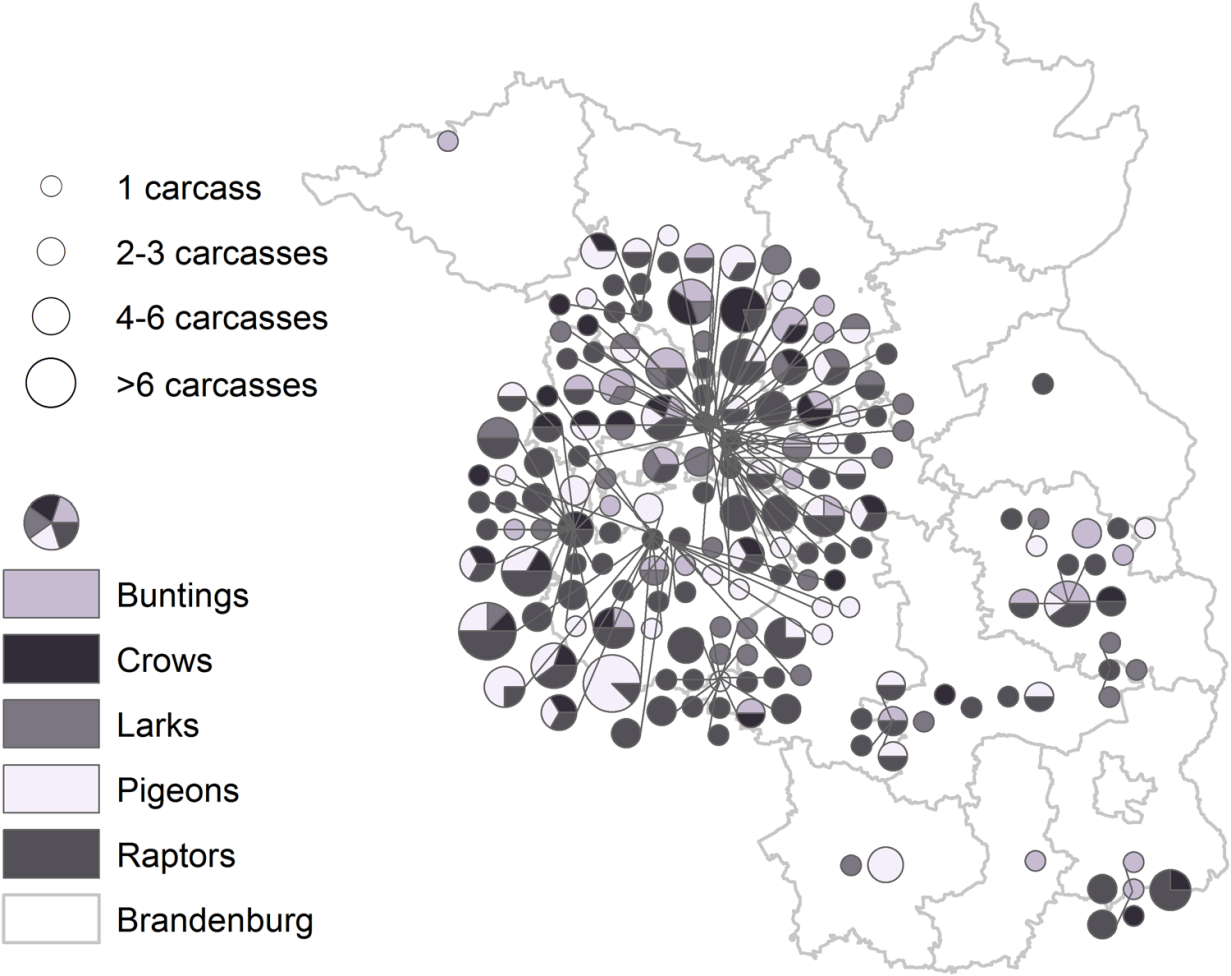
Relative abundance of the members of the worst hit bird-groups at the Wind Turbines with carcasses. With pies showing results of bird-group identifications expressed as relative frequencies (shading inside the pie), and total number of carcasses detected (size of the total pie) from each Wind Turbine. *ESRI*. *ArcGIS Desktop: Release 10*.*1*. *Redlands*, *CA: Environmental Systems Research Institute*.

We only used a subset of carcass detections for our analyses belonging to the following taxa (families): Buntings (*n* = 29), Crows (*n* = 30), Larks (*n* = 37), Pigeons (*n* = 55) and Raptors (*n* = 128) (Fig. 3). This taxonomical grouping criterion was chosen firstly because of morphological and ecological similarity and secondly with the aim to have sufficient individuals in the subsamples for statistical testing. Secondly, this taxonomic stratification followed was ultimately based on similar morphologies and ecological processes among the detected species. Such stratifications are based on linkages between taxonomic and functional diversities defined by firstly similarities in species morphologies that determine habitat and ability to colonize, followed by physiologies influencing their adaptiveness to the habitats based on rates and efficiencies of birth, death and resource utilization^24^ influencing their collision response at the WT structures. A very few species belonging to one of the chosen groups, e.g. the Kestrel (*Falco tinnuculus*) that belongs to the order of Raptors (Accipitriformes), but shows a bigger difference with respect to ecological aspects like attraction to urban and technical structures (high buildings, chimneys) unlike other species belonging to the same order. However, in this case all 12 detected Kestrels were found near to turbines that had also fatalities of other Raptor species (Red Kite *Milvus milvus* or White-tailed Sea Eagle *Haliaaetus albicilla* or both; 5 turbines) or that were not farther away than 300 to 500 meters from those (7 turbines). Therefore, a (substantial) bias caused by these recoveries can be excluded. Besides, all other single species counts scattered over other taxonomical groups were excluded from the analyses.

### Data preparation

#### Distance to edge based land-use variables (DELV)

The detailed database of land-use provided by the Biotope Type and Land Use Mapping Project of the State of Brandenburg of 2011^25^ was processed using the inclusive features at the level of the 12 major land-use classes (Table 1), avoiding the greater degrees of inconsistencies and lack of information associated with the succeeding subordinate classes. The different types of land-use classes were separated, features of the individual land-use classes were transformed to polylines and pre-processed individually for the creation of Euclidean distances at a cell resolution of 100 m for the whole study area using ArcGIS version 10.1^26^. The resolution of 100 m was chosen to find a compromise between accuracy, size of the raster maps, and available computer memory respectively processing time. Also, recommendations to policymakers are based on a resolution that is rarely higher than 100 m. For the ease of interpretation, the created Euclidean distances were prefixed with a negative or a positive sign, denoting distances inside or distances outside of the feature of a particular land-use class (Annex: Figure A1). For using these distances based land-use variables in *Biomapper 4*.*0* software http://www.unil.ch/biomapper^27^ we converted the ArcGIS generated ESRI grids into IDRISI raster formats.

**Table 1:** Distance to edge based land-use variables (DELVs) used as predictors in the Federal State of Brandenburg, Germany.

Variable	Description	Coverage (%)	Variable Acronym
Bushlands	Deciduous bushes, field bushes, tree-lined roads, tree groups and riparian woods	0.79	B
Fields	Plow lands, arable lands and other farmlands	35.11	F
Forests_forestry	Forests and commercial forests	35.51	FF
Flowing_watercourses	Streaming waters, springs, small flowing rivers and channels	0.39	FW
Green_areas_settlements	Biotopes of green areas and open spaces including parks, gardens and village greens	1.66	GS
Grass_forbs	Meadows, pastures, grasslands, lawns and forb areas	16.37	GF
Ruderal_areas	Anthropogenic raw soil sites and ruderal areas with or without very few vegetation	0.26	RA
Shrublands	Dwarf shrubs, heathlands and conifer bushes	0.35	S
Special_biotas	Special biotopes including valleys, plantations, commercial gardens and tree nurseries	0.87	SB
Settlements_structures	Buildings, roads, paths, traffic and industrial areas, railroads and village like developments	5.73	SS
Still_watercourses	Still waters, lakes, small waterbodies, reservoirs, ponds and mine waters	2.21	SW
Wetlands	Mosses, swamps, sedges and peat cutting sites	0.73	W

### Data analysis

#### Ecological-niche factor analysis

With the multivariate approach of ENFA, based on Hutchison’s niche theory^18,19^ we determined the collision sensitive ecological niche (for the sake of brevity called collision niche). We used turbines (with and without collisions) as sampling points. Surveyed turbines without collisions served as controls. Sampling points are thus restricted to the existing turbines in the landscape; firstly giving insights into the structure of landscape suitable for turbine installations (under existing policies e.g. places with strong steady wind, places away from forests, places away from settlements etc.). Amongst these sampling points some points have collisions, giving insight not only regarding the structures of the landscape that are suitable for turbine installations under current policies, but additionally to those factors more likely leading to collisions. Our study uses the spatial information of the turbines with detected bird carcasses, where presence^28^ in a grid cell is given the value of 1, and absence in a grid cell (no turbine or no turbine with detected carcasses) is given the value 0. This Boolean response map covering the whole study area acts like a mask that is analyzed in *Biomapper 4*.*0* software for ENFA against the gridded maps of the predictor variables to elucidate the combination of distances to different land-use types that lead to an increased risk of collision with WTs.

We specifically evaluated these combinations of distances for the worst hit taxon related groups of birds in our sample (Buntings, Crows, Larks, Pigeons and Raptors), focusing on similarities and dissimilarities between these bird-groups.

To enable the guidance of potential management interventions, we used ENFA based on Hutchinson’s *n*-dimensional hypervolume^18^ in a little different way. ENFA normally condenses the overall information into two indices; the first index is ‘marginality’ of the focal species, defined as the ecological distance between the species optimum and the mean habitat within the reference area^29^. In our special case, it maximizes the multivariate distance between the predictor variables for the cells with detected collisions and the predictor variables for all cells without turbines or collisions within the reference area. This index provides information about the extent to which the species collision sensitive niche differs with respect to the combinations of distances to different land-use types from that of the most frequent set of combinations available in the entire spatial multivariate reference set of the study area^30^ (Fig. 2). In our study, global marginality values closer to 1 will signify that there is a substantial difference with respect to the combination of distances between the composition and configuration of the study area as compared to the composition and configuration of the collision sensitive niche. Contrarily, a value closer to 0 will imply no difference^29^.

The second and the following indices are the ‘specialization’ indices. They maximize the specialization of a focal species, defined as the ratio of the ecological variance in mean habitat to that observed for the focal species^29^. The values account for the decreasing specialization in subsequent order, and denote the extent of the species distribution width with respect to the overall distribution of conditions in the reference area^30^. The inverse of specialization is a measure of the species tolerance^28^ to conditions that are increasingly distinctive from their optimum. In our study, species with greater specializations will have lower tolerance and their collision sensitivity at WTs will substantially increase only when its placement meets special combinations of distances based on spatial relations between different land-use types that promote collisions.

### Data analysis

#### Niche differentiation and overlap

We firstly used linear discriminant analysis (LDA) to discriminate among turbines with the detections of birds belonging to the different taxon related bird-groups and the turbines with no detected bird carcasses, independent of the niche concept. LDA was conducted using the MASS package^31^ in R^32^.

Secondly, we used the discriminant factors from the ENFA following the niche concept to make discriminant analyses using *Biomapper 4*.*0* software, between (pairs of) the bird-groups and compare the distribution of predictor variables amongst the groups simultaneously. This procedure then computes a factor maximizing the difference between the groups while minimizing the intra-group variance^33^. The resulting discriminant factors are therefore basically linear combinations of the predictor variables, with their coefficients identifying every variable’s contribution to discriminate the collision sensitive niche between each pair of the bird-groups under consideration. Hence, the discriminant scores highlight the variables for which the pair of groups in question differ the most.

Thirdly, the scores of their respective discriminant factors from the ENFA analyses were also used to compute indices quantifying their respective collision niche breadths and to assess their similarities on the basis of pairwise niche overlap analyses. The discriminant factor from one of the group is used and interpreted in the form of signs indicating the direction towards the first or the second species in comparison. The Hurlbert index (*B′)* was used to measure the niche breadths^34^, where *B′* ranges from 0 (corresponding to specialized niche) to 1 (corresponding to generalized niche)^35^. Lloyd’s asymmetric overlap index *(Z*) was computed to assess the extent of niche overlaps between the groups, where larger *Z* values and a smaller associated reciprocal *Z* value for a given pair of species signify greater niche overlap^34^ by the former on the latter. And lastly, the first discriminant factor from each of the respective ENFA of the bird-groups were also used to visually represent the respective predictor variables based conditions favoring collisions in the landscape of Brandenburg.

#### Distribution distances and comparison between turbines where fatalities were registered and those where no fatalities were found so far

We have investigated the group-wise significant differences between the distribution of the WTs with fatalities and the WTs without fatalities against different DELVs using the Kolmogorov-Smirnov test^36^, using the maximum vertical deviation between their respective cumulative distributions as the statistic ***D*** and their respective ***P***-values reporting the significance of difference. The hypothesis regarding the difference in their distributional form is rejected if ***D*** is greater than the critical value based on a table for the chosen significance level or if the directly calculated ***P***-value is smaller than the chosen significance level.

Although it has advantages of being non-parametric and making no assumptions about the distribution of the data^36^, there are practical issues as well when applying the Kolmogorov-Smirnov test to fatality search data, primarily due to low detectability, either due to being rapidly scavenged or due to being moderately vulnerable to the collision phenomena. Not taking into account such effects can lead to artificial results of small numbers that ultimately leads to wrong conclusions about the result of the Kolmogorov-Smirnov test, which we do consider in the discussion of the results.

### Data availability

All data files created in this study are available from the Dryad Digital Repository: 10.5061/dryad.j1h2v.

### Ethics Statement

The material for this study is based on a database registering counts and locations of birds found dead as a consequence of collision with WTs in the area of the Federal State of Brandenburg and (with a lesser intensity) whole Germany. This database only contains information of counts and carcasses registered in a special data sheet provided by the Brandenburg State Agency for Environment (Brandenburg State Bird Conservation Centre, Unit N3, Buckower Dorfstraße 34, 14715 Nennhausen/OT Buckow, Germany).

*Access:* http://www.lugv.brandenburg.de/cms/media.php/lbm1.a.3310.de/meldebogen_anflugopfer.xls.

More information about the sampling, the availability, and the results of analyses can be found at: http://www.lugv.brandenburg.de/cms/detail.php/bb1.c.312579.de.

No living animal was caught or killed for this study or for the collation of the used database. All animals collected and registered in this database were killed by collision with WTs independent of the sampling process and before the sampling was performed. The database contains data about protected and endangered species. However, these species were not killed or manipulated for this study, they were either found dead or seriously injured after their collision, registered and provided to the authority responsible for their registration and, if still possible, overhanded to an appropriate clinic for health treatment and release. There was either no permission necessary for the registration and collection of the carcasses on public land or the collection was performed in accordance with the requested monitoring or a special permission was either given by the hunter (if the species was game), the land owner or the nature conservation authority. All permissions followed the respective legislation at the federal and state levels. No illegal information is stored in this database. The database is hosted by the State Agency, which is deputized in this study by one of the authors (Tobias Dürr).

A detailed description of the content and availability of the databases is given in: *Dürr*, *T*. *(accepted): Bewertung und Nutzung der Schlagopferdatei als Hilfsmittel zur Analyse anlagebedingter Mortalität von Vögeln an Windenergieanlagen*. *(Assessment and use of data sets of bird strikes for the analysis of bird mortality at WTs)*. *In Derschke*, *V*., *Bernotat*, *D*. *& Grunewald*, *R*.*: “Bestimmung der Erheblichkeit und Beachtung von Kumulationswirkungen in der FFH-Verträglichkeitsprüfung”*. *BfN-Skripten*.

Available soon from the Internet pages of the Federal Agency for Nature Conservation: https://www.bfn.de/0306_eingriffsregelung-literatur.html/, https://www.bfn.de/0502_skripten+M52087573ab0.html.

## Results

### Ecological-niche factor analysis

Corresponding to the set of value combinations based on the land-use distance variables, the global marginality values for Crows (*M* = 1.17), Larks (*M* = 1.18), Buntings (*M* = 0.98), Raptors (*M* = 0.98) and Pigeons (*M* = 0.99) (Table 2). All bird-groups showed a similar degree of specializations [Buntings (*S* = 2.40), Crows (*S* = 2.54), Larks (*S* = 2.43), Pigeons (*S* = 2.29)], except for Raptors with a substantial lower value of specialization (*S* = 1.82) (Table 2). As global tolerance is the reverse of specialization, the collision tolerance at WTs for Buntings and Larks will be lower as compared to that of Raptors. Table 3 shows the relative influences of each predictor variable on the marginality and specialization factors for the five bird-groups at WTs (See Annex: Table A1 for the associated coefficient values of these factors), representing the influence of the respective predictor variables on the collision sensitivity that increases the risk for the birds to collide with a WT.

**Table 2:** Collision marginality (M) and specialization (S) values for the worst hit bird-groups at wind turbines in the Federal State of Brandenburg, Germany.

Bird-Groups	Marginality (*M*)	Specialization (*S*)
Buntings	0.98	2.54
Crows	1.17	2.40
Larks	1.18	2.43
Pigeons	0.99	2.29
Raptors	0.98	1.82

Marginality represents the extent of how different the group’s collision habitat is from the mean conditions available in the study area; an increasing M indicates increasing marginality. Specialization S represents the breadth of the collision prone niche for each group, with S > 1 indicating some degree of specialization.

**Table 3:** Contribution of the 12 predictor variables to the *m*arginality and *s*pecialization factors of the ENFA, of the worst hit bird-groups at *wind turbines* in the *F*ederal *S*tate of Brandenburg, Germany.

Marginality						Specialization				
	Buntings	Crows	Larks	Pigeons	Raptors	Buntings	Crows	Larks	Pigeons	Raptors
Eigenvalues	1.15	1.14	1.10	1.21	1.13	0.33	0.33	0.38	0.29	0.34
Specialization accounted for by the factor	Factor 1 (15%)	Factor 1 (14%)	Factor 1 (11%)	Factor 1 (22%)	Factor 1 (13%)	Factor 2 (34%)	Factor 2 (33%)	Factor 2 (38%)	Factor 2 (30%)	Factor 2 (34%)
Bushlands	++	+++	+++	++	++	*	**	**	*	*
Fields	−−−−^1^	−−−−^1^	−−−−^1^	−−−−^1^	−−−−^1^	******	***	********	****	********
Forests_forestry	++++++	+++++	++++++	+++++	+++++	**	0	*	**	***
Flowing_watercourses	+	0	+	0	+	*****	*******	****	***	****
Green_areas_settlements	+++	++++	+++	++++	++++	0	0	*	***	**
Grass_forbs	+++	++++	++++	++++	+++	*	*	*	**	*
Ruderal_areas	++	++	++	++++	++	***	*	*	***	*
Shrublands	++	+	++	+	+	**	****	*	**	**
Special_biotas	0	+	−1	−1	0	**	*	***	***	0
Settlements_structures	+	++	+	+++	++	**	**	*	***	*
Still_watercourses	+	++	+	++	++	**	*	**	*****	*
Wetlands	+++	+++	++	+	+++	*	**	*	**	*

Marginality factor 1−+: the focal bird-groups were detected at locations with values higher than the average cell value for the particular predictor variable, i.e. avoidance; −: an increasing negative distance may be understood as preferring proximity for the particular predictor variable. Specialization factor 2−*: the focal bird-groups occupied a narrower range of values for the particular predictor variable than those available in the reference set. The greater the number of symbols (+, −, *) the narrower the range; with each symbol reflecting an influence of 0.10 on a scale between 0 and 1 (+ = 0.1, ++++++++++ = 1), where 0 indicates a very weak correlation/low expression of the respective factor.

#### Raptors

The raptor marginality factor only accounted for 13% of the total sum of eigenvalues of the factors. The coefficients of arable lands loaded substantially to both axes, marginality and especially specialization (F1 = −0.41 and F2 = −0.79; Annex: Table A1) indicating strong evidence for the discovery of Raptor carcasses at distances closer to or even inside of fields and other arable lands. Their marginality coefficients also showed correlations to distances away from forests and forestry areas (0.50), green and open spaces outside human settlements (0.40) and grassland and forb areas (0.33), with the loadings for distances farther from forests being higher than the distances inside the fields. Likewise, their first specialization factor provided further insights on their collision niche breadth, being spanned mainly between distances farther from shrub-lands and distances inside the fields, with the weight more on the latter. In this factor, the variance in the sample of points, described by the turbines where fatalities of Raptors were found, is 1/16 the variance found in the sample of all other points in the study area. The coefficients showing the relation to the distances from fields and arable lands suggest that the distances to the edges of this particular land-use type has a major impact in limiting collision risk for Raptors.

#### Crows and Larks

The specialization factor for Crows accounted for 33% and that of the Larks accounted for 38% of the total sum of eigenvalues of factors, both illustrating high levels of specializations towards distances to flowing watercourses and arable lands, respectively. The niche is not very marginal (1.14, 1.10) for Crows and Larks, respectively, and in the same range as the one of the Raptors. However, the variance in the sample of turbines with fatalities for Crows and Larks is 1/12 and 1/6.5 of the variance found for all other points in this landscape. Their marginality coefficients showed similar preferences to all predictor variables, i.e. discovery of their carcasses showed correlations with the distance closer to fields and arable lands (−0.37 and −0.40, respectively) and with the distance away from forests and forestry areas (0.47 and 0.58, respectively) and grassland and forb areas (0.41 and 0.43, respectively) as well. In their first specialization factor, the variance in their sample of turbines is substantially smaller than the rest of the study area, with a ratio of 1:31.64 and 1:29.45, respectively.

#### Buntings

Buntings marginality and specialization factor accounted for 15% and 34% of the total sum of eigenvalues of factors, respectively, also indicating strong relationship with distances closer to fields and other arable lands (Fields coefficient F1 = −0.44 and F2 = −0.64; Annex: Table A1). In contrast to other bird-groups, the marginality factor of Buntings indicated that their carcass detections were more strongly influenced by distances away from forests and forestry areas (0.64) than by distances away from grasslands and forb areas (0.34) and green and open areas around human settlements and bush-lands (0.26).

#### Pigeons

The marginality factor of Pigeons accounted for only 22% of the total sum of eigenvalues of factors, with marginality coefficients indicating that Pigeon detections increased with the distances from forests and commercial forests (0.47), green and open spaces outside human settlements (0.36), and grassland and forb areas (0.38). The marginality and specialization axes (available = 11.348 and 21.641, respectively) indicated strong relationships with distances to fields and other arable lands (Fields coefficient F1 = −0.45 and F2 = −0.36; annex: Table A1).

**Figure 4:**
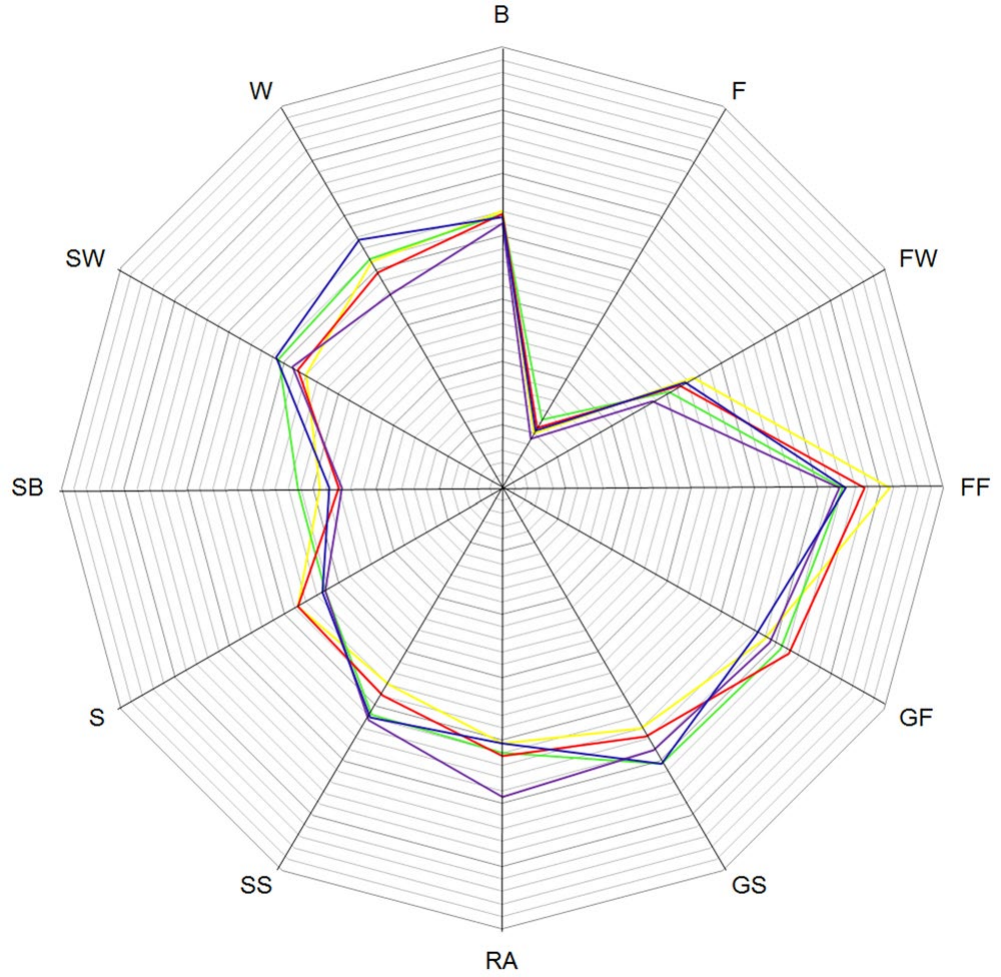
Collision sensitive niche positioning based on marginality coefficients (eigenvectors) ascertained by ENFA of the worst hit bird-groups at wind turbine structures in the study area. The colors yellow, green, red, purple and blue denote Buntings, Crows, Larks, Pigeons and Raptors, respectively. Acronyms corresponding to the predictor variables are described in Table 1.

The ratio of specialization accounted for by the first specialization factor for every bird-group suggests that the effects on their niche breadth were largely influenced by their respective factor coefficients, but the marginality factor (the ratio of the variance at all sample points versus the variance of the samples at turbines with carcasses) for Raptors accounted for less specialization (4.86:1) than for the Larks (6.57:1), Buntings (8.91:1), Pigeons (11.35:1) or Crows (11.79:1), indicating that they displayed a more restrictive range than Raptors for those conditions for which they differed from the mean of the study area (Fig. 4).

### Niche differentiation and overlap

**Figure 5:**
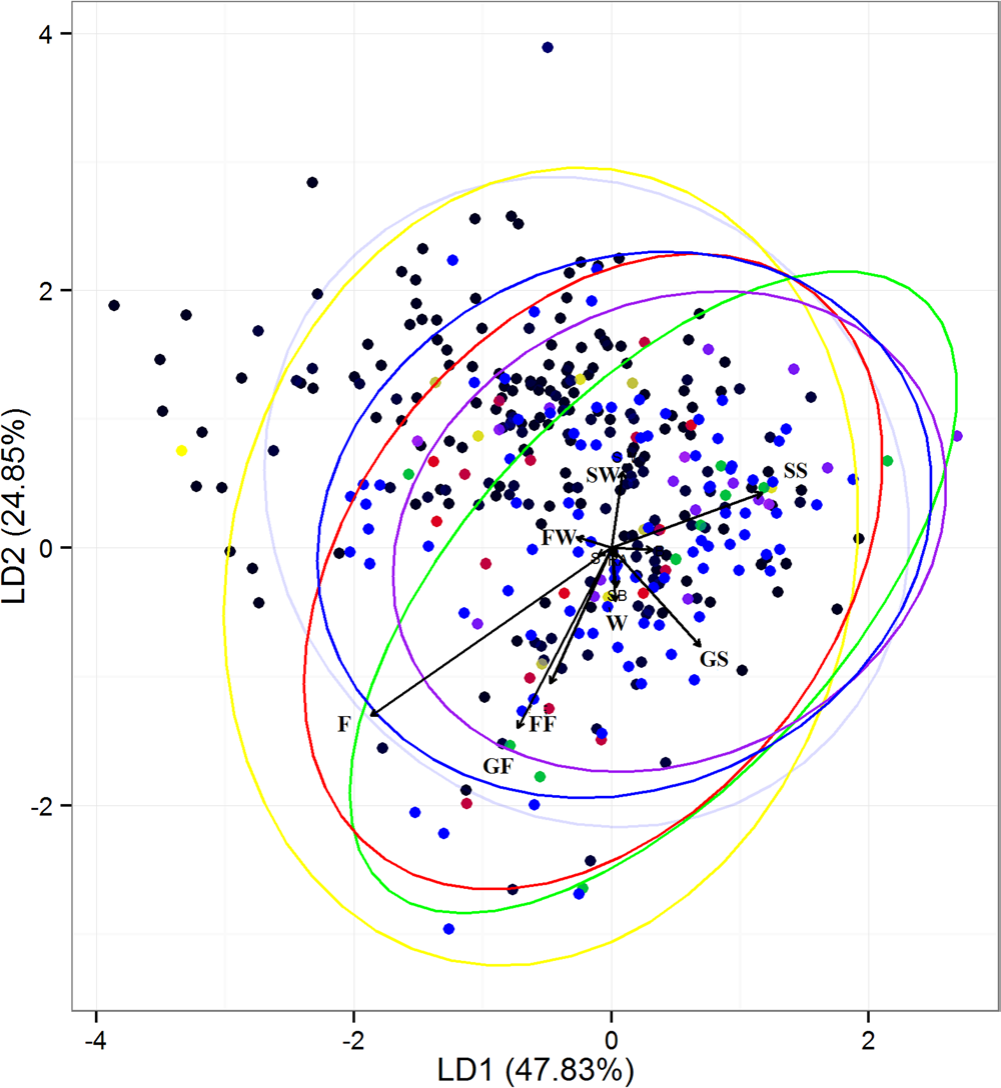
Linear discriminant analysis of the predictor variables representing the collision - no collision space showing the placement of the worst hit bird-groups at wind turbine structures in the study area. Black denotes no detections of collisions; yellow, green, red, purple and blue denote the bird-groups of Buntings, Crows, Larks, Pigeons and Raptors, respectively. Acronyms corresponding to the predictor variables are described in Table 1. (Please refer to Annex: Table A3 for the other variables and information regarding their respective influence on the axes)

The Linear Discriminant Analysis (LDA) provided weak discrimination among the location of WTs without detected carcasses and those with detected carcasses of the five worst hit bird-groups (Fig. 5). The first two linear discriminant axes (LD1 and LD2) together explained 25–48% of among-group variance in the LDA (Annex: Table A3). LD1 was positively influenced by distances to still watercourses and negatively influenced by distances to forests and forestry areas. LD2 on the other hand was strongly influenced by distances to flowing watercourses.

Hulbert’s niche breadth index indicated that the turbines where Raptors had collided showed a more general and greater expanse along landscape distance variables compared to that of the other groups (Table 4). This is also consistent with the results of the LDA (Fig. 5).

**Table 4:** Hulbert’s niche breadth index (B′) for the worst hit bird-groups at wind turbines in the Federal State of Brandenburg, Germany.

Bird-group	Hulbert’s Niche Breadth *B*′
Raptors	0.41
Pigeons	0.36
Larks	0.32
Buntings	0.30
Crows	0.26

B′ may range from 0–1, with 0 and 1 corresponding to specialists and generalists, respectively.

Lloyd’s asymmetrical niche overlap index consistently showed significantly greater overlap of the common collision space by the turbines with Raptor detections, followed by that of the Pigeon detections, especially on the detections of other bird-groups that have insignificant reciprocal overlaps on the former groups. The lowest overlap index was observed for the overlap of turbines with Bunting detections (Table 5).

**Table 5:** Lloyd’s asymmetrical overlap indices (Z) for the collision sensitive niches of the worst hit bird-group at the wind turbines in the Federal State of Brandenburg, Germany, and their reciprocals.

Niche Overlap	Raptors	Pigeons	Crows	Larks	Buntings
Raptors	—	9.34	5.74	7.38	4.71
Pigeons	18.48	—	5.87	7.57	4.93
Crows	17.80	9.20	—	7.86	4.06
Larks	19.62	10.17	6.74	—	5.02
Buntings	16.84	8.92	5.38	7.67	—

The small Z values, and larger associated reciprocals for each of the bird-groups with that of the group of Raptors, signifying greater niche overlap by the latter group. Rest combinations have almost similar overlaps on each-other i.e. equivalent.

### Discriminant Analysis

Supporting the results of niche differentiation and overlaps, the pairwise discriminant analyses between simultaneous pairs of each of the bird-groups also highlighted very low separation of their collision space. The predictor variables that still highly influenced the fundamental separations between the collision spaces of most of the group pairs are provided in (Annex: Table A2. Positive values (≥0.2) indicate variables that primarily contribute to the collision sensitive niche of the first bird-group of the pair, negative values (≤−0.2) to that of the second bird-group of the pair (Annex: Figure A2).

### Differences in Distance Distributions

The comparison of distributions of distances found for turbines where fatalities were registered with those where no fatalities were found so far gave additional insights regarding the importance of the different land-use classes for the bird-groups under investigation are shown in Table 6 and Figure A3. For the Raptors the distributions shifted significantly between distributions of turbines with carcasses as compared to turbines without carcasses, mainly for five land-use classes (flowing watercourses p = 0.000 (towards shorter distances), still watercourses p = 0.045 (towards farther distances), green areas around settlements p = 0.000 (towards farther distances), shrublands p = 0.045 (towards farther distances), and settlement and structures p = 0.030) (towards farther distances)), while for Pigeons they were found for three land-use classes (flowing water courses p = 0.000 (towards shorter distances), grassland and forb areas p = 0.038 (towards farther distances), ruderal areas p = 0.003 (towards farther distances)). Only two albeit different land-use classes were significantly different for Larks (grassland and forb areas p = 0.013(towards farther distances), Shrublands p = 0.004 (towards farther distances)), Crows (flowing watercourses p = 0.004 (towards shorter distances), green areas around settlements p = 0.006 (towards farther distances)) and Buntings (forests and forestry areas p = 0.016 (towards farther distances), special biotas p = 0.002 (towards farther distances)) respectively. The differences exist not only in median values but also in the extent and partly skewness of the distributions as can be seen from Figure A3 in the annex.

**Table 6:** Results of the comparison of distance distributions with the Kolmogorov-Smirnov test found for turbines without and turbines with fatalities for the worst hit bird-groups with regards to the predictor variables.

		Bush-lands	Fields	Forest_forestry	Flowing_water-courses	Green_areas_settlements	Grass_forbs	Ruderal_areas	Shrub-lands	Specialbiotas	Settlementstructures	Still_water-courses	Wetlands
Buntings	*D*	0.094	0.143	0.297	0.201	0.123	0.099	0.173	0.297	0.359	0.215	0.158	0.153
	*p*	0.970	0.629	0.016*	0.218	0.805	0.952	0.385	0.016	0.002**	0.158	0.501	0.548
Crows	*D*	0.144	0.190	0.206	0.334	0.322	0.190	0.304	0.201	0.291	0.156	0.153	0.167
	*p*	0.601	0.259	0.180	0.004**	0.006**	0.258	0.011	0.204	0.017	0.496	0.526	0.411
Larks	*D*	0.150	0.157	0.226	0.000	0.128	0.270	0.249	0.299	0.148	0.077	0.138	0.112
	*p*	0.425	0.369	0.060	1.000	0.625	0.013*	0.028	0.004**	0.437	0.987	0.528	0.782
Pigeons	*D*	0.102	0.157	0.112	0.393	0.097	0.201	0.257	0.158	0.164	0.158	0.147	0.158
	*p*	0.691	0.177	0.575	0.000***	0.748	0.038*	0.003**	0.175	0.143	0.172	0.239	0.174
Raptors	*D*	0.053	0.075	0.059	0.294	0.238	0.079	0.098	0.141	0.136	0.149	0.142	0.113
	*p*	0.952	0.662	0.894	0.000***	0.000***	0.589	0.321	0.045*	0.061	0.030*	0.045*	0.181

Significance levels *< = 0.05, **< = 0.01, ***< = 0.001.

## Discussions

The guidelines of the EU Habitats and Bird Directives make provisions to ensure the protection of wildlife against WT structures and recommend wind projects to be preceded by impact assessment studies and succeeded with post-construction (baseline) collision monitoring programs to determine impacts on wildlife at the project sites^37^. We used long-term collision detections from wind farms in the state of Brandenburg for the assessment of the worst hit groups of birds at WTs – Buntings, Crows, Larks, Pigeons, and Raptors. The main intent behind our examination was to assess to which particular land-use types and at what distances to these land-use types do WTs promote or reduce the collision risk. Distances are often required when policymakers ask for information ensuring safe deployment of WTs. Therefore, the results can be helpful in showing the increase and decrease of the collision risk at distances in the immediate vicinity or distant away from specific land-use types, thereby facilitating proposing safer placements of WTs in the landscape. Therefore, we analyzed the carcass detections in relation to the local landscape, specifically against the distances between and within multiple land-use types to the WT sites, to ascertain special combinations of distances leading to a higher risk of bird collisions.

The marginality coefficients for each group depict strong relationships between the turbines where carcasses have been detected and the following key land-use types: fields and other arable lands, forests and forestry areas, green and open areas outside human settlements and grassland and forb areas. With increasing or decreasing absolute values, signifying proximity with respect to the sign (inside−, outside+; announcing the direction). It is noteworthy that the proximity of the detections (group-wise) to particular land-use types on which our collision sensitive niche analyses (group-wise) are based, are alike.

The marginality factor of the data from Raptors further suggested higher importance of distances between turbines and green, open areas in and around human settlements as well as distances of turbines to forests. These findings are reflected in their observed carcass detections at turbines closer but outside the borders of forests and forestry areas up to distances of 2000 m (Annex: Figure A3) and is in line with expectations based on Raptor proximities to forests and forestry areas that provide them with suitable nesting and breeding places^38,39^. Our results are also in accordance with the minimum distances of wind turbines to breeding sites of Raptors as recommended by the Working Group of German State Bird Conservancies, based on species-specific telemetry studies, collision data, functional-spatial analyses, long-term observations and expert assessments, taking into account the risk of collision, avoidance and barrier effects caused by wind turbines^40^.

Raptors are also highly abundant in the fringe zones of infrastructures^41^, primarily due to adequate hunting options^42^, especially of many human-commensal small mammals^43–45^ and the availability of roadkill carrions^46^, with observed carcasses at turbines situated from their borders between 400–2400 m distances (Annex: Figure A3). They are also observed using features of the urban landscape, such as trees adjacent to open covers, fences and buildings, as shelter from wind, pollution, domestic predators, and concealment in ambush attacks on their prey and for purposes of perching, utilizing new and artificial nesting substrates^47–50^. Pigeons likewise, another abundant bird species in built-up environments, have also adapted their nesting requirements and foraging habits to be conducive with the urban lifestyles^51^ and particularly, green and open areas and urban parks surrounding heavily urbanized areas, settlements and infrastructures have higher densities of these species, as they take advantage of food discarded by humans favoring a more stable presence^52^, explaining the increase in Pigeon carcass detections at turbines closer to their borders, with detection primarily observed between 1000 m and up to 1700 m (Annex: Figure A3).

The marginality and specialization factorial axes of all the bird-groups also indicate strong relationships with distances to arable lands, highlighting their impact in limiting their collision sensitive niches. In case of Raptors, their associations with certain elements of the agricultural landscapes, especially arable lands and open fields, is primarily because of hunting facilitated by mowing or use of low-stature crops^53^, exposing preys to aerial predators^54^. Moreover, the fallow land at the mast foot provide suitable small-mammal habitat in the agricultural landscape, irrespective of low- or high-stature crops^55^. Placement of WTs generally has to follow many criteria; the site under consideration should have a strong potential for wind and should neither be near to settlements nor to areas of important habitats for birds or protected species that could be harmed^56^. With the reluctance of local people to install WTs near their homes, project developers often attempt to install wind energy facilities on agricultural land, particularly on arable land dominated by open fields^57^. These areas are also characterized by large plots of grassland or large fields of crops. Therefore, we can find almost all of the already constructed WTs inside of fields or open grasslands. This spatial preference also adds on to the ecological affinities certain bird-groups, particularly Larks show towards open landscapes. They avoid tall, dense vegetation cover^58^, and nest and forage in open agricultural fields, that influences most of their habitat preferences and reproductive success^59,60^, which in turn increases their risk of colliding with the turbine structures closer to the borders of fields, grasslands and open areas. With carcasses detected near to wind turbines situated between −400 m up to 100 m distances from the borders of fields and majorly detected between 300 m and 700 m distances from the borders of grasslands and open areas (Annex: Figure A3).

ENFA results also show that Raptors have the lowest global specialization value in comparison to the other bird-groups and also a comparatively larger niche breadth as per Hulbert’s niche breadth analysis. The ENFA analyses and the LDA analyses also denote that the coverage of the collision space by Raptors is larger compared to that of the other bird-groups, explaining their asymmetrical niche overlap with the other bird groups. Raptors have a greater home range^61,62^ as compared to many other birds of smaller size, and venture across distances to utilize perch and prey availability^49^. This indicates that the greater Raptor overlap is either an effect of the comparably larger parameter space covered by the Raptors or a better coverage of the detections in the study area because of their larger sample size, i.e. the exceptionally high number of Raptor carcasses detected at WTs in comparison to other smaller birds. This is primarily due to higher searcher efficiencies in combination with longer carcass persistence times for Raptors^13^.

The least observed niche overlaps based on turbine sites where collisions were detected show that the rather restrictive collision niche of Buntings has an insignificant overlap with the collision niches of other bird-groups, especially Crows. Crows being generalist omnivores^63^ and Buntings being shrub-land specialists^64,65^, mostly show niche differentiations on grounds of their specific preferences towards proximity to green and open areas in and around settlements and proximity to shrub-lands respectively. This is in accordance with our pairwise discriminant analysis, showing turbines with Bunting and Crow detections having fundamental niche separations related to the distances to the edges of shrub-lands (favoring Bunting detections) and green areas around human settlements (favoring Crow detections). These results are also consistent with ENFA, where Buntings show higher global specialization values as compared to other groups.

Overlaps of the respective collision niches of the bird-groups indicate similar sensitivities of birds to the multiple land-use combinations, whereas niche differentiations indicate the reverse. Niche overlap is often used to indicate potential for competition between species^33,35,66^. However, in this study, with respect to renewable energy infrastructure the overlaps between species provides insights into their similar or disparate sensitivities to distances from different land-use types that allow directing safer turbine positioning for protecting multiple bird-groups at once as well as for targeting specific groups with limited overlaps with other groups.

## Conclusion

Using the simplistic ordination procedure of ENFA, based on presence-absence of WT hit bird carcasses; we found that individuals of the worst hit group of birds in the state of Brandenburg showed an appreciable extent of overlaps between their collision spaces. Raptors showed the greatest overlaps with all other groups, most likely due to their broad range, covering the parameter space of the reference area as well as their appreciably greater probability to be hit by the turbine structures and be detected afterwards owing to their bigger body sizes that have greater persistence times and are easier to detect. Moreover, despite of the fact that our study was only based on carcass detections, it gave a detailed descriptive analysis of the turbines with collisions with respect to their placement distances to land use types. Although our method is not suitable for predictions of the impacts on and viability of bird populations, the detected greater Raptor niche overlaps compared to the other groups indicate that Raptors may serve as a suitable proxy for birds in general for purposes of impact assessments and be a safer starting point to develop and test theories in an experimental framework to better understand the relationship between landscape compositions and the risks to birds from technical infrastructures for wind energy production. Such studies will not only pave the way for future research but also enable improved guidance for management interventions and the spatial allocation of wind farms to serve the transition to renewable energies while keeping impacts on species minimal.

## Electronic supplementary material

Supporting information
