# Supplementary material for: Collision sensitive niche profile of the worst affected bird-groups at wind turbine structures in the Federal State of Brandenburg, Germany
Source: Sci Rep. 2018 Feb 28;8:3777. doi: 10.1038/s41598-018-22178-z (PMC5830649; doi:10.1038/s41598-018-22178-z)

# Supporting information – Supplementary tables and graphs

Collision sensitive niche profile of the worst affected bird-groups at wind turbine structures in the federal state of Brandenburg, Germany

Anushika Bose[1]*, Tobias Dürr[2], Reinhard A. Klenke[1&] and Klaus Henle[1&]

[1]UFZ – Helmholtz Centre for Environmental Research, Department of Conservation Biology, Permoserstraße. 15, D-04318 Leipzig, Germany

[2] Brandenburg State Agency for Environment, Brandenburg State Bird Conservation Centre, Unit N3, Buckower Dorfstraße 34, 14715 Nennhausen/OT Buckow, Germany

* Corresponding author

E-mail: anushika.bose@ufz.de (AB)

**Supporting information:**

**Annex: Table A1: Relevant first five out of 12 axes of the ENFA with their eigenvalues (in brackets) and the predictor variable coefficients of the worst hit bird-groups at wind turbines in the Federal State of Brandenburg, Germany.**

**Annex: Table A2: Discriminant factor coefficients between the collision environmental envelopes between every pair of the worst hit bird-groups at wind turbine structures in the Federal State of Brandenburg.**

**Annex: Table A3: Linear discriminant coefficients and proportion of variance explained by each axis.**

**Annex: Figure A1**: **Distance to edge presentations of land-use variables in the Federal State of Brandenburg.** *R Development Core Team. 2013. R: A language and environment for statistical computing. R Foundation for Statistical Computing, Vienna, Austria. ISBN 3-900051-07-0. http://www.R-project.org*

**Annex: Figure A2: Predictor variables discriminant coefficients between the collision environmental envelopes of every pair of the worst hit bird-groups at wind turbine structures in the Federal State of Brandenburg.**

**Annex Figure A3**: **Distance distributions of turbines without (A, C, E, G and I) and with fatalities (B, D, F. H, and J) for the worst hit bird-groups with regards to the predictor variables: Raptors, Pigeons, Larks, Crows and Buntings respectively.**

**Annex: Table A1: Relevant first five out of 12 axes of the ENFA with their eigenvalues (in brackets) and the predictor variable coefficients of the worst hit bird-groups at wind turbines for the Federal State of Brandenburg, Germany**

The positive or negative sign is relevant for the first axis coefficients, but in the following axes only the absolute value of coefficients is considered. The greater the absolute value of the marginality coefficient, the more this variable contributes to the group's marginality factor. Absolute values ≥ 0.40 are considered strongly influential. The +/- prefixes of the predictor variable require a flip in interpretation of these strongly influential variables. The negative coefficients indicate that the distances of the land-use type to the collision sensitive niche was less than what was globally available in the study area, and the positive coefficients indicate that the collision sensitive niche for the particular bird-group was farther away from the land-use type than the average distance available across the sample area. The percentages in parentheses indicate the amount of total specialization accounted for by each factor.

| | | Marginality | Specialization | | | |
|---|---|---|---|---|---|---|
| | **Variables** | **Factor 1 (8.913)** | **Factor 2 (28.076)** | **Factor 3 (11.445)** | **Factor 4 (7.262)** | **Factor 5 (6.301)** |
| | **Bushlands** | 0.22 | 0.11 | -0.05 | 0.30 | -0.38 |
| | **Fields** | -0.44 | -0.64 | -0.03 | 0.04 | 0.33 |
| | **Forests_forestry** | 0.64 | 0.18 | -0.04 | -0.02 | 0.13 |
| | **Flowing_watercourses** | 0.11 | 0.49 | -0.64 | -0.30 | -0.23 |
| | **Green_areas_settlements** | 0.26 | 0.00 | -0.01 | -0.37 | 0.59 |
| **Buntings** | **Grass_forbs** | 0.34 | 0.08 | 0.08 | -0.14 | -0.34 |
| | **Ruderal_areas** | 0.20 | 0.32 | 0.04 | 0.49 | 0.14 |
| | **Shrublands** | 0.15 | -0.24 | -0.06 | -0.09 | 0.34 |
| | **Special_biotas** | -0.02 | -0.25 | -0.66 | 0.45 | 0.17 |
| | **Settlements_structures** | 0.08 | 0.19 | 0.21 | 0.26 | 0.11 |
| | **Still_watercourses** | 0.13 | 0.21 | -0.20 | -0.25 | 0.14 |
| | **Wetlands** | 0.25 | -0.05 | 0.26 | 0.29 | 0.12 |

| | Variables | Factor 1 (11.786) | Factor 2 (31.638) | Factor 3 (18.218) | Factor 4 (7.910) | Factor 5 (6.662) |
|---|---|---|---|---|---|---|
| **Crows** | **Bushlands** | 0.26 | -0.20 | 0.00 | 0.35 | 0.15 |
| | **Fields** | -0.37 | -0.31 | 0.60 | -0.39 | -0.35 |
| | **Forests_forestry** | 0.47 | -0.03 | 0.17 | -0.15 | -0.23 |
| | **Flowing_watercourses** | 0.02 | -0.74 | -0.44 | -0.27 | 0.02 |
| | **Green_areas_settlements** | 0.42 | 0.02 | -0.05 | -0.35 | -0.01 |
| | **Grass_forbs** | 0.40 | -0.15 | 0.02 | 0.15 | -0.23 |
| | **Ruderal_areas** | 0.24 | -0.14 | 0.31 | -0.06 | 0.21 |
| | **Shrublands** | 0.06 | 0.39 | 0.02 | -0.43 | 0.13 |
| | **Special_biotas** | 0.05 | -0.14 | 0.17 | -0.23 | 0.65 |
| | **Settlements_structures** | 0.24 | -0.18 | 0.27 | 0.15 | 0.22 |
| | **Still_watercourses** | 0.22 | 0.15 | -0.33 | -0.44 | -0.47 |
| | **Wetlands** | 0.25 | 0.20 | 0.32 | 0.15 | -0.02 |

| | Variables | Factor 1 (6.567) | Factor 2 (29.454) | Factor 3 (14.899) | Factor 4 (8.476) | Factor 5 (6.520) |
|---|---|---|---|---|---|---|
| Larks | **Bushlands** | 0.26 | 0.16 | 0.05 | 0.04 | 0.20 |
| | **Fields** | -0.40 | 0.76 | -0.09 | 0.16 | 0.10 |
| | **Forests_forestry** | 0.58 | 0.12 | -0.32 | -0.04 | 0.06 |
| | **Flowing_watercourses** | 0.05 | -0.43 | -0.24 | 0.71 | -0.24 |
| | **Green_areas_settlements** | 0.29 | 0.08 | -0.35 | -0.30 | -0.08 |
| | **Grass_forbs** | 0.43 | 0.13 | -0.02 | 0.00 | -0.07 |
| | **Ruderal_areas** | 0.25 | 0.13 | 0.28 | 0.36 | 0.39 |
| | **Shrublands** | 0.16 | 0.12 | 0.64 | -0.10 | -0.30 |
| | **Special_biotas** | -0.08 | -0.28 | -0.15 | -0.30 | 0.65 |
| | **Settlements_structures** | 0.14 | 0.07 | 0.12 | 0.33 | 0.13 |
| | **Still_watercourses** | 0.14 | 0.20 | -0.14 | -0.18 | -0.35 |
| | **Wetlands** | 0.20 | 0.12 | 0.40 | 0.06 | 0.27 |

| | Variables | Factor 1 (11.348) | Factor 2 (21.641) | Factor 3 (9.493) | Factor 4 (6.858) | Factor 5 (3.862) |
|---|---|---|---|---|---|---|
| **Pigeons** | **Bushlands** | 0.22 | -0.05 | 0.07 | -0.14 | 0.18 |
| | **Fields** | -0.45 | -0.36 | 0.61 | -0.15 | -0.05 |
| | **Forests_forestry** | 0.47 | -0.18 | 0.11 | -0.06 | -0.32 |
| | **Flowing_watercourses** | -0.04 | -0.25 | -0.45 | -0.61 | -0.01 |
| | **Green_areas_settlements** | 0.36 | -0.28 | 0.20 | 0.12 | -0.26 |
| | **Grass_forbs** | 0.38 | -0.24 | 0.05 | -0.05 | 0.44 |
| | **Ruderal_areas** | 0.36 | 0.28 | 0.33 | -0.28 | 0.21 |
| | **Shrublands** | 0.05 | 0.24 | 0.15 | 0.37 | 0.43 |
| | **Special_biotas** | -0.08 | 0.33 | 0.34 | -0.26 | 0.08 |
| | **Settlements_structures** | 0.27 | 0.33 | 0.05 | -0.23 | -0.51 |
| | **Still_watercourses** | 0.16 | -0.49 | -0.24 | 0.47 | 0.14 |
| | **Wetlands** | 0.12 | 0.20 | 0.24 | -0.12 | 0.29 |

| | Variables | Factor 1 (4.861) | Factor 2 (15.558) | Factor 3 (5.998) | Factor 4 (4.942) | Factor 5 (3.328) |
|---|---|---|---|---|---|---|
| Raptors | Bushlands | 0.24 | 0.06 | -0.02 | 0.15 | -0.19 |
| | Fields | -0.41 | -0.79 | -0.32 | 0.09 | 0.11 |
| | Forests_forestry | 0.50 | -0.28 | -0.08 | -0.17 | -0.07 |
| | Flowing_watercourses | 0.07 | -0.35 | 0.86 | -0.02 | 0.16 |
| | Green_areas_settlements | 0.40 | -0.24 | 0.01 | 0.03 | 0.40 |
| | Grass_forbs | 0.33 | -0.13 | 0.04 | -0.02 | -0.19 |
| | Ruderal_areas | 0.20 | -0.11 | -0.19 | 0.40 | -0.15 |
| | Shrublands | 0.06 | 0.24 | -0.17 | 0.24 | 0.74 |
| | Special_biotas | -0.04 | -0.01 | 0.07 | 0.60 | -0.02 |
| | Settlements_structures | 0.24 | -0.10 | -0.11 | 0.10 | -0.11 |
| | Still_watercourses | 0.22 | -0.08 | -0.02 | -0.50 | 0.33 |
| | Wetlands | 0.31 | 0.06 | -0.26 | 0.32 | -0.15 |

**Annex: Table A2: Discriminant factor coefficients between the collision environmental envelopes between every pair of the worst hit bird-groups at wind turbine structures in the Federal State of Brandenburg.** Positive values (≥0.2) indicate variables favor the collision environmental envelope of the first bird-group of the pair, and the negative values (≤ -0.2) favorable that of the latter.

| DELV | Bunting & Crows | Buntings & Larks | Buntings & Pigeons | Buntings & Raptors | Crows & Larks | Crows & Pigeons | Crows & Raptors | Larks & Pigeons | Larks & Raptors | Pigeons & Raptors |
|---|---|---|---|---|---|---|---|---|---|---|
| **Bushlands** | -0.199 | 0.171 | -0.017 | 0.006 | -0.231 | 0.201 | -0.281 | -0.185 | 0.028 | 0.106 |
| **Fields** | -0.217 | -0.22 | -0.247 | -0.228 | -0.196 | 0.487 | 0.073 | -0.121 | -0.019 | -0.307 |
| **Forests_forestry** | 0.152 | -0.129 | -0.293 | 0.232 | 0.032 | -0.109 | -0.116 | 0.181 | -0.029 | 0 |
| **Flowing_watercourses** | 0.225 | -0.387 | -0.315 | 0.236 | 0.328 | 0.3 | 0.427 | 0.379 | -0.495 | -0.321 |
| **Green_areas_settlements** | -0.391 | 0.104 | 0.183 | -0.457 | -0.193 | -0.055 | -0.379 | -0.008 | -0.383 | -0.047 |
| **Grass_forbs** | -0.054 | 0.228 | -0.034 | -0.009 | 0.373 | 0.115 | -0.105 | 0.512 | -0.227 | -0.04 |
| **Ruderal_areas** | -0.332 | 0.324 | 0.391 | -0.164 | -0.201 | -0.378 | -0.485 | -0.199 | -0.023 | 0.581 |
| **Shrublands** | 0.455 | 0.283 | -0.347 | 0.345 | 0.209 | -0.206 | 0.05 | 0.219 | 0.059 | -0.063 |
| **Special_biotas** | 0.141 | -0.323 | -0.411 | 0.409 | 0.011 | 0.034 | -0.182 | -0.098 | 0.235 | 0.005 |
| **Settlements_structures** | -0.398 | 0.338 | 0.499 | -0.366 | -0.262 | -0.236 | -0.125 | -0.299 | -0.102 | 0.224 |

| | | | | | | | | | | |
|---|---|---|---|---|---|---|---|---|---|---|
| **Still_watercourses** | 0.238 | -0.117 | -0.016 | -0.382 | 0.559 | 0.473 | 0.364 | 0.552 | -0.558 | -0.574 |
| **Wetlands** | -0.362 | 0.525 | 0.166 | -0.191 | -0.398 | -0.372 | -0.383 | -0.143 | 0.417 | 0.258 |

**Annex: Table A3: Linear discriminant coefficients and proportion of variance explained by each axis.**

| Coefficients | LD1 | LD2 | LD3 | LD4 | LD5 |
|---|---|---|---|---|---|
| **Bushlands** | 0.00032 | -0.00002 | 0.00065 | 0.00024 | 0.00033 |
| **Fields** | -0.00190 | -0.00130 | -0.00200 | 0.00055 | -0.00140 |
| **Forests_forestry** | -0.00047 | -0.00100 | -0.00044 | 0.00033 | -0.00021 |
| **Flowing_watercourses** | -0.00027 | 0.00008 | -0.00013 | 0.00009 | 0.00022 |
| **Green_areas_settlements** | 0.00069 | -0.00076 | -0.00120 | 0.00022 | 0.00059 |
| **Grass_forbs** | -0.00073 | -0.00140 | 0.00063 | 0.00210 | 0.00110 |
| **Ruderal_areas** | 0.00004 | -0.00007 | 0.00002 | -0.00003 | 0.00001 |
| **Shrublands** | -0.00010 | -0.00006 | -0.00002 | -0.00001 | -0.00017 |
| **Special_biotas** | 0.00004 | -0.00030 | -0.00005 | -0.00047 | 0.00020 |
| **Settlements_structures** | 0.00120 | 0.00043 | -0.00051 | 0.00043 | -0.00180 |
| **Still_watercourses** | 0.00009 | 0.00059 | 0.00023 | 0.00068 | 0.00027 |
| **Wetlands** | 0.00003 | -0.00041 | -0.00030 | -0.00011 | -0.00026 |
| ***Proportion of group variance*** | ***0.48*** | ***0.25*** | ***0.16*** | ***0.07*** | ***0.04*** |

**Annex: Figure A1**: **Distance to edge presentations of land-use variables in the Federal State of Brandenburg.**

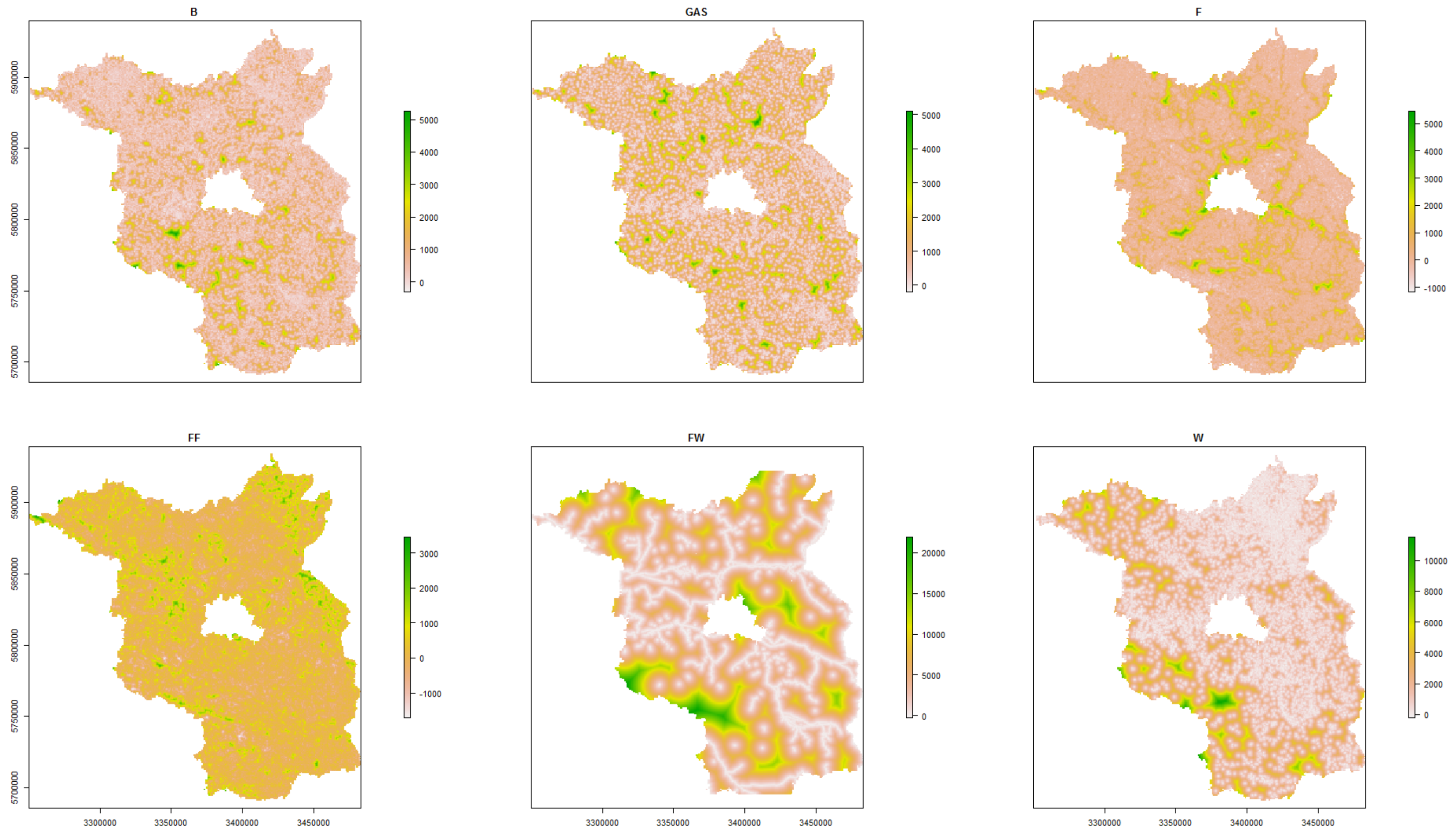


[1]Acronyms corresponding to the predictor variables are described in Table 1.

**Annex: Figure A2: Predictor variables discriminant coefficients between the collision environmental envelopes of every pair of the worst hit bird-groups at wind turbine structures in the Federal State of Brandenburg.**

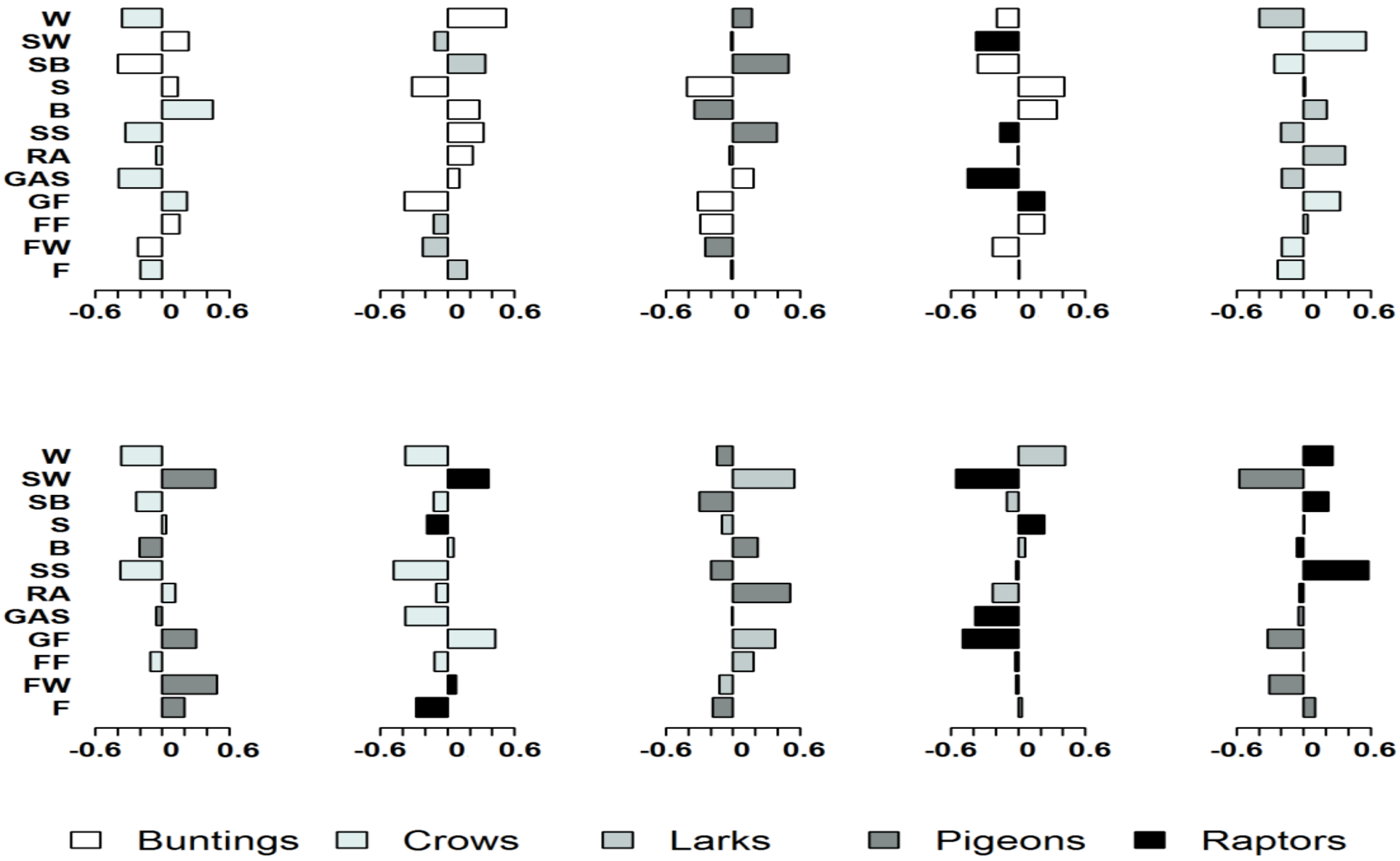

**Annex: Figure A3: Distance distributions of turbines without (A, C, E, G and I) and with fatalities (B, D, F. H, and J) for the worst hit bird-groups with regards to the predictor variables:** Raptors, Pigeons, Larks, Crows and Buntings respectively.

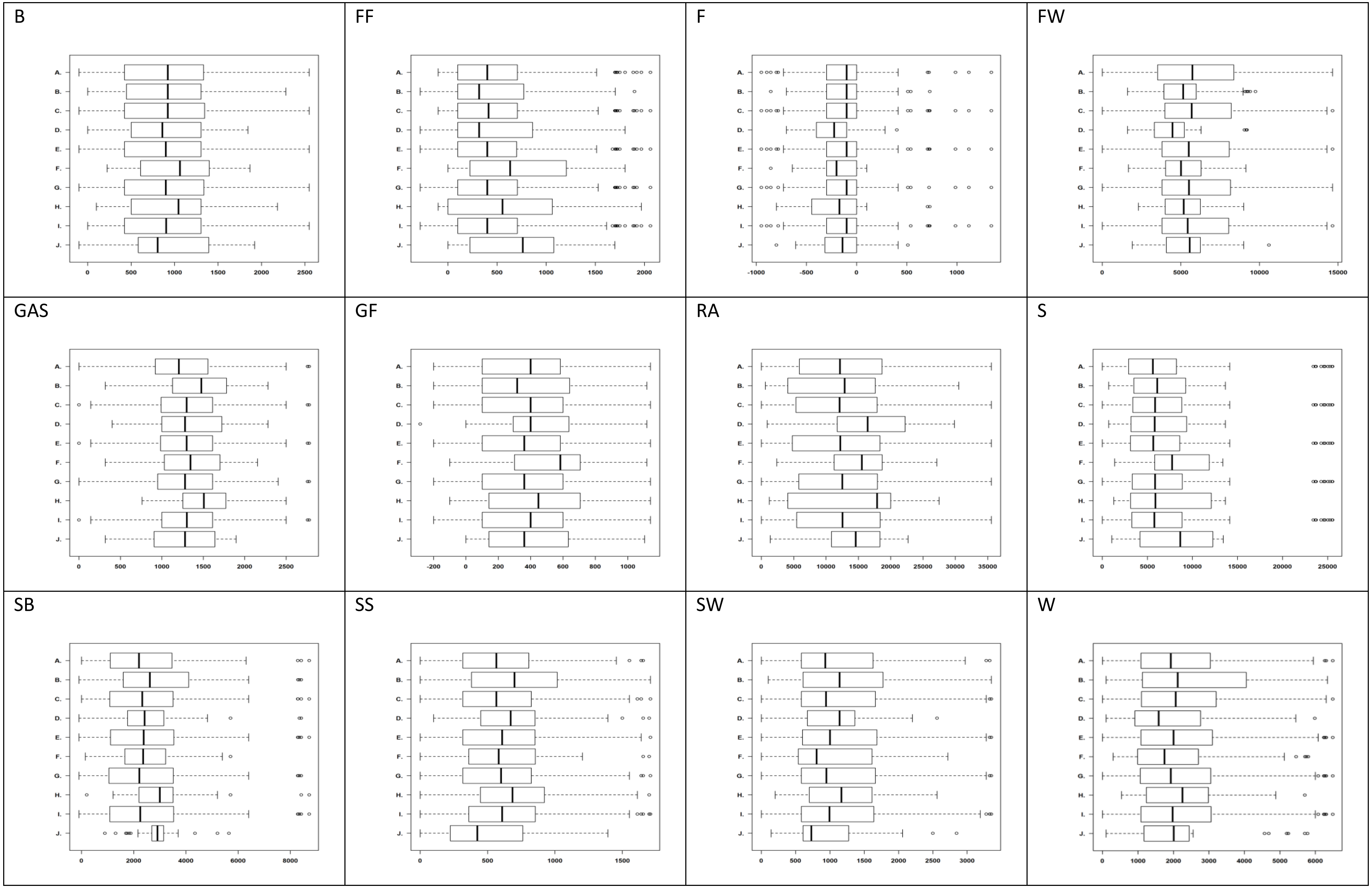

Supplement: Supplementary file 1 — Supporting information [file 41598_2018_22178_MOESM1_ESM.pdf]
